# Ethical and Legal Concerns Associated With Withdrawing Mechanical Circulatory Support: A U.S. Perspective

**DOI:** 10.3389/fcvm.2022.897955

**Published:** 2022-07-26

**Authors:** Paul S. Mueller

**Affiliations:** Department of Medicine, Division of General Internal Medicine, Mayo Clinic Health System, La Crosse, WI, United States

**Keywords:** mechanical circulatory support, ventricular assist device, total artificial heart, extracorporeal membrane oxygenation, medical ethics, advance care planning, palliative care, end of life

## Abstract

Hundreds of thousands of Americans have advanced heart failure and experience severe symptoms (e. g., dyspnea) with minimal exertion or at rest despite optimal management. Although heart transplant is an effective treatment for advanced heart failure, the demand for organs far exceeds the supply. Another option for these patients is mechanical circulatory support (MCS) provided by devices such as the ventricular assist device and total artificial heart. MCS alleviates symptoms, prolongs life, and provides a “bridge to transplant” or a decision regarding future management such as “destination therapy,” in which the patient receives lifelong MCS. However, a patient receiving MCS, or his/her surrogate decision-maker, may conclude ongoing MCS is burdensome and no longer consistent with the patient's healthcare-related values, goals, and preferences and, as a result, request withdrawal of MCS. Likewise, the patient's clinician and care team may conclude ongoing MCS is medically ineffective and recommend its withdrawal. These scenarios raise ethical and legal concerns. In the U.S., it is ethically and legally permissible to carry out an informed patient's or surrogate's request to withdraw any treatment including life-sustaining treatment (LST) if the intent is to remove a treatment perceived by the patient as burdensome and not to terminate intentionally the patient's life. Under these circumstances, death that follows withdrawal of the LST is due to the underlying disease and not a form of physician-assisted suicide or euthanasia. In this article, frequently encountered ethical and legal concerns regarding requests to withdraw MCS are reviewed: the ethical and legal permissibility of withholding or withdrawing LSTs from patients who no longer want such treatments; what to do if the clinician concludes ongoing LST will not result in achieving clinical goals (i.e., medically ineffective); responding to requests to withdraw LST; the features of patients who undergo withdrawal of MCS; the rationale for advance care planning in patients being considered for, or receiving, MCS; and other related topics. Notably, this article reflects a U.S. perspective.

## Introduction

Hundreds of thousands of Americans have advanced heart failure and experience severe symptoms (e.g., dyspnea) with minimal exertion or at rest despite optimal management (e.g., lifestyle changes, medications, devices, and surgery). The median time from the diagnosis of advanced heart failure to death is 12 months (1, 2). Diagnosing advanced heart failure is important as affected patients may be eligible for heart transplant and/or mechanical circulatory support (MCS), which alleviate symptoms and prolong life (1).

However, in the U.S., only about 3,000 hearts are transplanted each year and many heart transplant candidates die waiting for donor hearts (3). While on the waiting list, some heart transplant candidates receive MCS with a surgically implanted ventricular assist device (VAD), a scenario known as “bridge to transplant.” A VAD, which is connected to a control system and an energy source outside of the body, pulls blood from the left ventricle and pumps it into the aorta. Patients with potentially reversible heart failure can be supported with a VAD while waiting for their hearts to regain function, a scenario known as “bridge to recovery.” Some patients with permanent advanced heart failure who are not heart transplant candidates can be supported with a VAD indefinitely, a scenario known as “destination therapy” (4).

Similarly, some patients with heart failure may be eligible for MCS provided by a total artificial heart (TAH). Implantation of a TAH requires removal of most of the recipient's native heart. A pneumatically driven diaphragm directs blood through two mechanical ventricles; four mechanical valves ensure unidirectional blood flow. Drive lines connect the TAH to an air compressor outside of the body. The choice between VAD and TAH is determined by the patient's underlying pathophysiology. Indications TAH include severe biventricular heart failure, heart transplant graft failure, cardiac malignancy, infiltrative or restrictive cardiomyopathies, congenital heart disease, and others. TAH is used for “bridge to transplant” and “destination therapy” (5).

Extracorporeal membrane oxygenation (ECMO) is another form of MCS. With ECMO, blood is drained from the venous system and pumped through a semipermeable membrane allowing for oxygenation and removal of carbon dioxide. The blood is then reinfused into the venous system (venovenous ECMO) or the arterial system (venoarterial ECMO) depending on the patient's underlying pathophysiology. For example, patients with respiratory failure, but normal cardiac function, may receive venovenous ECMO; the patient's heart circulates ECMO-treated blood. Patients with cardiac or cardiopulmonary failure may be receive venoarterial ECMO; the ECMO pump circulates blood independent of the patient's underlying heart function. ECMO is usually provided in intensive care units. ECMO scenarios include “bridge to transplant” (heart and/or lung), “bridge to MCS” (e.g., VAD), “bridge to recovery,” and “bridge to decision” for patients whose clinical situations are unclear (6).

The prevalence of Americans receiving MCS with VADs, TAHs, and ECMO is increasing. Also, these technologies are improving (e.g., size, ease of use, outcomes, etc.). Nonetheless, morbidity (e.g., infection, stroke, and multiorgan failure) and mortality in patients receiving MCS are substantial (7). A patient receiving MCS, or his/her surrogate decision-maker, may conclude the treatment is burdensome and no longer consistent with the patient's healthcare-related values, goals, and preferences and, as a result, request withdrawal of MCS. Likewise, the patient's clinician may conclude ongoing MCS as medically ineffective and recommend its withdrawal. These scenarios raise ethical and legal concerns.

In this article, the following frequently encountered ethical and legal concerns regarding requests to withdraw MCS are reviewed: the permissibility of withholding or withdrawing life-sustaining treatments (LSTs) including MCS from patients who no longer want such treatments; what to do if the clinician concludes ongoing LST will not result in achieving clinical goals (i.e., medically ineffective); responding to requests to withdraw LST including MCS; the features of patients who undergo withdrawal of MCS; the rationale for advance care planning in patients being considered for, or receiving, MCS; and other related topics. Notably, this article reflects a U.S. perspective.

## Ethical and Legal Precedents Regarding Withholding and Withdrawing Life-Sustaining Treatments and Implications for Clinical Practice

### *Prima facie* Principles of Ethics

Clinical ethics involves identifying, analyzing, and resolving moral problems that arise while caring for patients (8). Four *prima facie* principles encompass most ethical dilemmas encountered while caring for patients. *Beneficence* is the duty to act in the best interests of the patient. *Non-maleficence* is the duty to avoid harming patients (including not providing ineffective treatments). *Respect for patient autonomy* is the duty to respect the patient's rights to bodily integrity and self-determination. *Justice* is the duty to treat the patient fairly. Sometimes these principles conflict with each other. For example, contemplating a patient's request to withdrawal MCS may conflict with the clinician's desire to help, and avoid harming, the patient (8, 9).

### Is It Ethically and Legally Permissible to Carry out a Patient's or a Surrogate's Requests to Withhold or Withdraw Life-Sustaining Treatment?

A life-sustaining treatment is one that prolongs life without which patient death would likely occur. There are many LSTs including mechanical ventilation, hemodialysis, artificial nutrition and hydration, MCS, and others. Ethically and legally, withholding and withdrawing treatment are equivalent (10, 11). Carrying out a request to withhold or withdraw any treatment, including LST, is predicated on informed consent and, specifically, informed refusal. The principle of respect for patient autonomy is the basis of informed consent and refusal. Patient autonomy is optimized when the patient understands his/her diagnosis and treatment options, including no treatment, and participates fully in decision-making regarding these options. The clinician is obligated to ensure the patient is informed regarding his/her diagnosis and treatment options. Components of informed consent and refusal include information (typically the amount of information a reasonable patient needs), patient voluntariness, and patient decisional capacity. Decisional capacity refers to the patient's ability to make healthcare-related decisions. Requirements for decisional capacity include being able to grasp pertinent information, understand the clinical situation at hand, rationally manipulate information, make a decision consistent with one's own healthcare-related values, goals, and preferences, and communicate a decision. The clinician should not presume decisional incapacity if the patient makes a decision contrary to the clinician's recommendation. Rather, capacity should be presumed. Nonetheless, evidence for capacity varies according to the complexity of the decision to be made; i.e., the more complex the decision to be made, the higher the level of capacity required to make it. Notably, in the U.S., “competence” is a legal term and determined by courts. Most patients who lose decisional capacity due to illness are not declared incompetent by courts. Rather, in these situations, clinicians determine decisional capacity. With rare exceptions (e.g., an emergency), the clinician should not treat the patient without informed consent. The patient has the right to accept a proposed treatment, proceed with another option, or refuse treatment altogether (10, 11).

In the U.S., codes of ethics are clear regarding the patient's right to make healthcare-related decisions. Not only does the patient have the right to refuse any treatment, he/she also has the right to refuse any ongoing previously consented treatment, including LST, if the patient concludes the burdens of the treatment outweigh the benefits and is inconsistent with his/her healthcare-related values, goals, and preferences. While the effectiveness of a treatment (e.g., based on clinical trials) is the purview of the clinician, the burdens and benefits of a treatment are the purview of the patient. Whatever the clinician's intent, commencing or continuing a treatment the patient does not want is battery. The clinician's duty is to ensure the patient's refusal of, or request to withdraw, a treatment is informed (10–13).

U.S. courts have consistently ruled that, based on rights to bodily integrity and self-determination, the patient has the right to make healthcare-related decisions including refusing LST before it is started and requesting its withdrawal after it is started. The precedents established by landmark U.S. court cases include: (a) the right to refuse, or request the withdrawal of, any treatment including LST; (b) there is no difference between withholding a treatment and withdrawing an ongoing treatment; (c) the patient refusing, or requesting the withdrawal of, LST need not be terminally ill; (d) carrying out an informed refusal of, or request to withdraw, LST is not a form of physician-assisted suicide or euthanasia; (e) death that follows carrying out an informed refusal of, or request to withdraw, LST is due to the underlying disease; (f) the patient without decisional capacity has the same rights through a surrogate decision-maker (see below); (g) no treatment has unique moral status in that the treatment must be started or, once started, must be continued; and (h) there is no right to physician-assisted suicide or euthanasia (10, 14–16). Parenthetically, consistent with the “rule of double effect,” clinicians should provide treatment to alleviate suffering even if the treatment has the potential to hasten patient death. Doing so, if the intent is to relieve suffering, is ethical, legal, and not a form of physician-assisted suicide or euthanasia (17–20) (Table 1).

**Table 1:** Precedents of landmark U.S. court cases regarding the permissibility of carrying out informed refusals of, or requests to withdraw, life-sustaining treatments.

1. Patients have a right to bodily integrity and self-determination; imposing treatment on a patient who does not want the treatment is battery
2. There is no difference between withholding a treatment and withdrawing an ongoing treatment
3. A patient has the right to refuse, or request the withdrawal of, any treatment including life-sustaining treatment a. The patient need not be terminally ill b. The clinician's duty is to ensure that a refusal of, or request to withdraw, treatment is informed c. Carrying out an informed refusal, or request to withdraw, life-sustaining treatment is not a form of physician assisted suicide or euthanasia d. Death that follows carrying out an informed refusal, or request to withdraw, life-sustaining treatment is due to the underlying disease
4. A patient without decisional capacity has the same rights as a patient who has decisional capacity through a surrogate
5. No treatment, including life-sustaining treatment, has unique moral status in that the treatment must be started or, once started, it must be continued
6. There is no right to physician-assisted suicide and euthanasia
7. Clinicians should provide treatment to alleviate suffering even if the treatment might hasten a patient's death (rule of double effect); the clinician's intent determines whether the act is a form of physician-assisted suicide or euthanasia

### Who Makes Healthcare-Related Decisions for the Patient When the Patient Cannot?

In the U.S., a court-appointed guardian makes medical decisions for the patient declared incompetent by the court. For the patient who lacks decisional capacity due to medical illness, clinicians must rely on a surrogate. If the patient has an advance directive (AD), and the AD identifies a surrogate, that person should make decisions for the patient (10, 11). An AD is a legal document completed by a patient that provides instructions for future care in the event the patient loses decisional capacity. In general, there are three types of ADs: health care power of attorney, living will, and combined ADs. In a health care power of attorney, the patient designates another person as his/her surrogate decision-maker. In a living will, the patient provides instructions for future care (e.g., LST) and circumstances (e.g., terminal illness). The patient can also indicate his/her healthcare-related values, goals, and preferences, what to do if pregnant, and organ donation. The combined AD has features of both a healthcare power of attorney and living will. Laws regarding ADs vary by U.S. state, but all 50 states regard the AD as an extension of the patient when he/she was fully autonomous (21).

The surrogate should make decisions based on the contents of the patient's AD. In addition, the clinician should adhere to the contents of the patient's AD, unless the instructions are unreasonable (e.g., impractical, illegal, etc.). There are benefits of having an AD. For example, a systematic review of 45 observational studies showed that patients who had ADs experienced reduced rate of hospitalization, reduced risk for dying in hospitals, reduced use of LSTs, and increased used of hospice and palliative care (22). Also, ADs coupled with advance care planning, may reduce moral distress among surrogates and clinicians when making difficult decisions by providing insights regarding the patient's healthcare-related values, goals, and preferences (i.e., what the patient would decide if he/she had decisional capacity).

Unfortunately, only about one-quarter of U.S. adults have ADs (21, 23). However, most U.S. states have laws which specify a hierarchy of surrogate decision-making for patients who lack decisional capacity and don't have ADs. Typically, a spouse, child, or other first-degree relative is the surrogate. Nonetheless, there is variability among U.S. states regarding these hierarchies (23).

When making decisions for the patient who lacks decisional capacity, the surrogate should adhere to the contents and instructions in the patient's AD, if extant. The surrogate should make decisions based on “substituted judgment”; i.e., based on the patient's, not the surrogate's, healthcare-related values, goals, and preferences. To optimize “substituted judgement,” a useful question to ask the surrogate is, “If (the patient) could wake up for 15 minutes, understand [his/her] current medical situation completely, and then had to go back into it, what would [he/she] tell us to do? (24)” If the patient's healthcare-related values, goals, and preferences are unknown, the surrogate should base his/her decisions on the “best interests” of the patient (e.g., quality of life). In the rare instance in which the clinician and/or care team perceive the surrogate is not making decisions for the patient based on substituted judgment or best interests, meeting with the surrogate to explore these concerns and ethics consultation should be considered (11).

Notably, although no treatment has unique moral status, some U.S. states necessitate high levels of evidence of the patient's wishes regarding artificial hydration and nutrition (e.g., written documentation in an AD) before carrying out a surrogate's request for it to be withdrawn (10).

### What Should Be Done if the Patient's Clinician Concludes Ongoing LST Will Not Result in Achieving Clinical Goals?

The American Medical Association Code of Ethics states, “Physicians are not ethically obligated to deliver care that, in their best professional judgment, will not have a reasonable chance of benefitting their patients” (11 p16). A clinician and his/her care team may conclude ongoing LST will not result in achieving clinical goals such as restoration of consciousness and discharge to home (i.e., medically ineffective). In these circumstances, the team should seek to understand the patient's healthcare-related values, goals, and preferences, obtain input from the surrogate if necessary, provide prognostic information, and take into account the intent of the LST, which the AMA declares “should not be to prolong the dying process without benefit to the patient…” (11 p18). The clinician and care team should consider the patient's cultural and religious beliefs and how these might affect decision-making regarding withdrawal of LST. Based on this information, the clinician and care team should recommend withdrawal of LST coupled with a shift to palliative care. This process can be facilitated by holding a multi-disciplinary care conference, which involves the patient (if able), surrogate, loved ones, clinicians, nurses, chaplain, and other care team members. Shared decision-making, consensus regarding withdrawal of LST, and clarifying goals of care should be emphasized. For some cases, ethics consultation can be helpful (10, 11).

### Is Withdrawing Mechanical Circulatory Support a Form of Physician-Assisted Suicide or Euthanasia?

Clinicians may wonder whether withdrawing MCS is a form of physician-assisted suicide or euthanasia. However, carrying out an informed request to withdraw an unwanted LST such as MCS differs from physician-assisted suicide and euthanasia in important ways. First, when withdrawing an unwanted LST, the clinician's intent is to remove a treatment the patient or surrogate regards as non-beneficial, burdensome, and inconsistent with the patient's healthcare-related values, goals, and preferences—not to hasten the patient's death. In contrast, in physician-assisted suicide, the clinician's intent is to terminate the patient's life by providing a lethal prescription to be taken by the patient. In euthanasia, the clinician's intent is to terminate the patient's life by administering a lethal agent to the patient. Second, death that follows carrying out a patient's or surrogate's informed request to withdraw an unwanted LST is due to the patient's underlying disease and pathophysiology. In contrast, in physician-assisted suicide and euthanasia, death that follows taking, or being administered, a lethal prescription is due to the lethal prescription—a newly introduced pathology—not the underlying disease (4, 5, 10, 15–17, 20).

The U.S. Supreme Court has differentiated withholding and withdrawing LST from physician-assisted suicide and euthanasia. In *Vacco*, Chief Justice Rehnquist wrote,

“The distinction comports with fundamental legal principles of causation and intent. First, when a patient refuses life-sustaining medical treatment, he dies from an underlying fatal disease or pathology; but if a patient ingests lethal medication prescribed by a physician, he is killed by that medication…our assumption of a right to refuse treatment was grounded not…on the proposition that patients have a…right to hasten death, but on well-established traditional rights to bodily integrity and freedom from unwarranted touching” (16).

While the U.S. Supreme Court has declared a constitutional right to refuse treatment, it has not declared a constitutional right to physician-assisted suicide or euthanasia. Today, physician-assisted suicide is illegal in most U.S. states and euthanasia is illegal in all states (10, 17).

In summary, carrying out an informed patient's or surrogate's request to withdraw LST is ethically and legally permissible and not a form of physician-assisted suicide or euthanasia (Table 2). While, to the author's knowledge, no U.S. court case has involved a patient receiving MCS, given the results of prior ethical analyses involving MCS (4–6) and court decisions involving other LSTs, the same conclusion can be drawn about carrying out informed requests to withdraw MCS.

**Table 2:** End of life interventions, causes of death, clinicians' intention of the interventions, and legality of the interventions in the U.S. From Olsen et al. (17).

	**Withhold life-sustaining treatment**	**Withdraw life-sustaining treatment**	**Palliative analgesia and sedation**	**Physician-assisted suicide**	**Euthanasia**
Cause of death	Underlying disease	Underlying disease	Underlying disease^a^	Intervention prescribed by the physician and used by the patient	Intervention administered by the physician
Intention of the intervention	Avoid burdensome intervention	Remove burdensome intervention	Relieve symptoms	Termination of the patient's life	Termination of the patient's life
Legality of the intervention?	Yes^b^	Yes^b^	Yes^c^	No^d^	No

a *Note the rule of double effect (18)*.

b *Some U.S. states limit the power of surrogates to make decisions about life-sustaining treatments*.

c *Washington v. Glucksberg (19)*.

d *Physician-assisted suicide is legal in several U.S. states*.

### Is Mechanical Circulatory Support a Morally Unique Treatment?

Despite the permissibility of carrying out informed requests to withdraw other LSTs, MCS has features that may cause some clinicians to question the permissibility of carrying out requests to withdraw it. For example, some argue MCS once started should not be withdrawn as it is a treatment that is continuous and constitutive (i.e., provides a vital function the patient's body no longer provides). Also, death inevitably follows withdrawal of MCS. However, it is widely accepted that carrying out informed requests to withdraw other continuous and constitutive LSTs (e.g., mechanical ventilation, artificial nutrition and hydration, etc.) is ethically and legally permissible (4–6, 25).

Some clinicians may view MCS as a replacement treatment (i.e., the treatment becomes part of the body and assumes all functions of the diseased organ) and, hence, cannot be withdrawn. However, a genuine replacement treatment is one that is responsive to physiologic changes and independent of external control, maintenance, and energy sources. Examples of replacement treatments include bioprosthetic heart valves and organ transplants. Carrying out a request to remove these treatments would be invasive, harmful, introduce a new pathology (i.e., surgical wound) and, hence, unethical. In contrast, MCS does not have the features of a genuine replacement treatment. Also, withdrawing MCS is noninvasive and painless (4–6, 25).

Some clinicians may object to carrying out requests to withdraw MCS as doing so may acutely precipitate heart failure symptoms (e.g., dyspnea) and death may occur shortly thereafter. However, similar symptoms and death may occur shortly after withdrawal of other LSTs such as intravenous inotropic agents, intra-aortic balloon pump therapy, and mechanical ventilation—treatments that are commonly withdrawn in end of life situations. Hence, the possibility of acute symptoms and death shortly after withdrawal of MCS are not ethically relevant (4–6). Rather, the clinician and care team should inform the patient or surrogate regarding potential symptoms associated with withdrawal of MCS, the likely timing of death, and once withdrawal occurs, manage the dying process expectantly.

As mentioned previously, U.S. courts have not recognized any treatment as being morally unique (10). However, courts have not considered withdrawal of MCS. Nonetheless, MCS does not have features that make it a morally unique treatment.

### What Are the Features of Patients Who Undergo Withdrawal of Mechanical Circulatory Support?

Three case series describing the features of patients who have undergone withdrawal of MCS have been reported by Mayo Clinic researchers. In a series of 68 patients who received VADs during 2003–2009, 26 had died, of which 14 requested, or surrogates requested, withdrawal of VAD support. All were receiving other LSTs. Eight patients died with multiorgan failure. For 12 patients, requests for withdrawal of VAD support were made by surrogates. For 11 patients, multidisciplinary care conferences were held before withdrawing VAD support. All died within a day of withdrawing of VAD support (4).

In a series of 47 patients who received TAHs during 2007–2015, 21 had died, of which 14 requested, or their surrogates requested, withdrawal of TAH support. All were receiving other LSTs. All died with multiorgan failure. For 13 patients, requests for withdrawal of TAH support were made by surrogates. For all 14 patients, multidisciplinary care conferences were held before withdrawing TAH support. All died within minutes of withdrawing TAH support (5).

In a series of 235 patients who received ECMO during 2010–2014, 118 had died. For 62 patients, requests for withdrawal of ECMO support were made. All were receiving other LSTs. Forty-six patients died with multiorgan failure. None of the patients had decisional capacity. For all patients, decisions to withdrawal ECMO were jointly reached by surrogates and clinicians. All died within a day of withdrawing ECMO support (6).

Other series have had similar results (26, 27).

Notably, in these three series, many patients did not have ADs, and of those who did, their ADs did not mention the MCS technology.

## Actionable Recommendations

### Responding to Requests to Withdraw Mechanical Circulatory Support: Who Decides? By What Criteria? How Are Conflicts Resolved?

Obviously, intentionally withdrawing MCS from a patient without consent is wrong and a form of killing. Because physician-assisted suicide is illegal in most U.S. states and euthanasia is illegal in all U.S. states, the clinician's intent when withdrawing MCS must be to remove a treatment the patient or surrogate regard as non-beneficial, burdensome, and no longer consistent with the patient's healthcare-related values, goals, and preferences. The intent must not be to terminate the patient's life (17).

Pellegrino describes a 3-question approach to responding to requests to withdraw LST (28). The first question is, “Who decides?” In the U.S., ethically and legally, the patient has the authority to make healthcare-related decisions and this authority supersedes the clinician's authority. If the patient lacks decisional capacity, then the patient's AD and surrogate guide decision-making. The clinician's duty is to ensure that a request to withdraw LST is informed. In the 3 previously described case series, only a few patients had decisional capacity. Hence, for most patients, surrogates made decisions. Of the patients who had ADs, none of the ADs addressed MCS. Hence, surrogates had to rely on “substituted judgement” or “best interests” in making decisions. For nearly all patients, decisions to withdraw MCS were made after multidisciplinary care conferences.

The second question is, “By what criteria?” Answering this question requires assessment of the LST's clinical effectiveness, and its perceived benefits and burdens. The clinician determines treatment effectiveness, whereas the patient determines treatment benefits and burdens. In the 3 previously described series involving MCS, nearly all decisions to withdraw MCS were made during multidisciplinary care conferences. Ongoing MCS was perceived by clinicians and care teams as medically ineffective and by patients and usually surrogates as non-beneficial and burdensome. This scenario, in which MCS merely maintains circulation and a moribund state, has been described as “destination nowhere” (29). In the three previously described series, withdrawal of MCS was justified.

The third question is, “How are conflicts among decision-makers resolved and prevented?” When conflicts arise in the care of patients receiving MCS, care conferences involving the patient, surrogate, and multidisciplinary care teams, as well as palliative care and ethics consultation can be helpful in resolving them. All patients being considered for, or receiving, MCS should undergo advanced care planning, also known as “preparedness planning,” including completion of an AD that addresses the MCS technology and its management at the end of life. Involving palliative medicine specialists in this process can be especially helpful. During these discussions, the circumstances surrounding permissibility of withdrawing MCS should be discussed including what to do if device failure, catastrophic complications, debilitative comorbid conditions (e.g., stroke), and inadequate quality of life occur (10, 11, 30–32). Such planning may prevent conflicts.

Processes for withdrawing specific MCS technologies—VAD, TAH, and ECMO support—are described elsewhere (33–37). Overall, withdrawal of MCS should be based on established palliative care principles and evidence-based best practices. The clinician should anticipate and manage symptoms that occur during the withdrawal process, involve palliative medicine specialists if necessary, and provide comfort to affected love ones.

### What if a Clinician Conscientiously Objects to Withdrawing Mechanical Circulatory Support?

Some clinicians, despite the ethical and legal permissibility of carrying out informed requests to withdraw LST, may object to the practice. If acceding to such a request violates a clinician's conscience, then the clinician should arrange for a transfer of the patient's care to another accepting clinician. In the meantime, the patient should not be abandoned. Similarly, clinicians and other care team members (e.g., nurses) who conscientiously object to withdrawing MCS should not be compelled to do so (4–6, 10).

## Conclusions

In the U.S., the prevalence of patients with advanced heart failure is increasing. Heart transplant is an effective treatment for patients with advanced heart failure. However, the demand for organs far exceeds the supply. In these patients, MCS alleviates symptoms, prolongs life, and “bridges” patients to transplant or a decision regarding future management such as “destination therapy” in which the patient receives lifelong MCS. However, the patient, or his/her surrogate, may determine that the burdens of ongoing MCS outweigh the benefits and is no longer consistent with the patient's healthcare-related values, goals, and preferences and, as a result, request withdrawal of MCS. Likewise, the patient's clinician and care team may conclude ongoing MCS is medically ineffective and recommend its withdrawal. In the U.S., it is ethically and legally permissible to carry out requests to withdraw LST made by an informed patient, or his/her surrogate, if the intent is to remove a burdensome treatment and not to terminate the patient's life. Under these circumstances, death that follows withdrawal of the LST is due to the underlying disease and not a form of physician-assisted or euthanasia. It is the clinician's duty to ensure that such requests are informed. These concepts also apply to withdrawal of MCS. Given the seriousness of his/her illness, the patient being considered for, or treated with, MCS should engage in advance care planning and document his/her healthcare-related values, goals, and preferences including end of life care and the management of the MCS device. Likewise, palliative care consultation should be considered for all such patients. When carrying out a request to withdraw MCS, clinicians should anticipate and manage symptoms that occur, involve palliative medicine specialists if necessary, and provide comfort to affected love ones.

## Author Contributions

The author confirms being the sole contributor of this work and has approved it for publication.

## Conflict of Interest

The author declares that this article was written in the absence of any commercial or financial relationships that could be construed as potential conflicts of interest.

## Publisher's Note

All claims expressed in this article are solely those of the authors and do not necessarily represent those of their affiliated organizations, or those of the publisher, the editors and the reviewers. Any product that may be evaluated in this article, or claim that may be made by its manufacturer, is not guaranteed or endorsed by the publisher.

## Abbreviations

LST: life-sustaining treatment
MCS: mechanical circulatory support
VAD: ventricular assist device
TAH: total artificial heart
ECMO: extracorporeal membrane oxygenation
AD: advance directive.
